# Rate of Improvement following Volar Plate Open Reduction and Internal Fixation of Distal Radius Fractures

**DOI:** 10.4061/2011/565642

**Published:** 2011-08-11

**Authors:** Chris Dillingham, MaryBeth Horodyski, Aimee M. Struk, Thomas Wright

**Affiliations:** Department of Orthopaedics and Rehabilitation, University of Florida, 3450 Hull Road, Gainesville, FL 32608, USA

## Abstract

*Purpose*. To determine recovery timeline of unstable distal radius fractures treated by open reduction and internal fixation with a locking volar plate. *Methods*. Data was collected prospectively on a consecutive series of twenty-seven patients during routine post-operative visits at 2 and 6 weeks, and 3, 6, 12 and 24 months. Range of motion measures and grip strength for both wrists were recorded. *Results*. Greatest gains were made within the first 3 months after surgery. Supination and pronation returned more quickly than flexion or extension, with supination and pronation both at 92% of the uninjured wrist at 3 months. Only flexion improved significantly between 3 and 6 months. All wrist motions showed some improvement until 1 year. Grip strength returned to 94% of the uninjured wrist by 12 months. *Conclusions*. Range of motion improvement will be greatest between 2 weeks and 3 months, with improvement continuing until 12 months. Grip strength should return to near normal by one year. Function and pain will improve, but not return to normal by the end of 12 months. *Clinical Relevance*. These results provide the surgeon with information that can be shared with patients on the anticipated timeline for normal recovery of function and strength.

## 1. Introduction

Distal radius fractures are the most common upper extremity fracture treated by orthopaedic surgeons. Abraham Colles originally described these fractures in 1814 prior to the advent of radiographs [1]. Since that time, treatment has changed significantly. Closed treatment was once widely advocated; however, with improved internal fixation devices and techniques, operative treatment has gained much support. Many distal radius fractures are displaced dorsally and tend to redisplace with conservative treatment [2]. A nonlocking dorsal buttress plate is ideal for fixation of these injuries. However, this method of fixation can result in a high complication rate secondary to tendon irritation [3]. With the advent of the locking plate fixation, treating these fractures from the volar aspect of the radius has gained popularity due to its ease of approach and decreased incidence of tendon irritation [1, 4]. Despite its increased use, the rate of return of motion in patients after operative treatment with volar plating has not been fully explored in the literature. The purpose of our study was to follow prospectively the rate of return of wrist motion and function in patients undergoing volar locked plate fixation for treatment of an unstable distal radius fracture. This is valuable information for the surgeon and will improve his ability to counsel patients preoperatively. Both patient and physician will have realistic expectations of recovery linked to a valid timeline.

## 2. Materials and Methods

Between August 2002 and October 2008, 27 patients treated for unstable displaced fractures of the distal radius were followed prospectively during and after open reduction and internal fixation with a volar fixed angled plate. Decision for surgery was made in all cases by the senior surgeon based on fracture pattern and degree of displacement. All surgeries were performed by the senior author. The local Institutional Review Board approved the protocol for this study before the study began. All subjects were informed of their rights under HIPAA and gave informed consent for their data to be used. The protocol conformed to ethical guidelines of the 1975 Declaration of Helsinki. All procedures were performed through a standard flexor carpi radialis volar approach using the same volar locked plate. 

Postoperatively, all patients were placed in a volar splint. At 2 weeks of followup the splint was removed. An active, passive, and active-assistive program was begun, supported by a removable splint. At 6 weeks postoperatively, the splint was discarded and strengthening was initiated. Data was collected at 5 follow-up visits within the first year. The primary outcome variables were wrist range of motion: flexion, extension, pronation, and supination. These measurements were collected at each visit by one of the certified hand therapists at our hand clinic. 

Secondary outcome measurements included Disabilities of the Arm, Shoulder, and Hand (DASH) scores, Patient-Rated Wrist Evaluation (PRWE) scores, and grip strength. This data was collected at 3, 6, and 12 month postoperative visits. In order for data to be included in the study, the patient had to have been seen at a minimum of three of the five visits without missing two consecutive visits. We had 24 months followup on 11 patients, too few for statistical analysis to be meaningful. 

Fifteen women and 12 men were included in the study. Their mean age was 53 years (SD = 18.4 years; range, 19–88 years). The dominant hand was involved in 14 patients and nondominant in 13 patients. Eighteen fractures were intra-articular; 9 were extra-articular fractures. The fractures were classified using the Müller AO classification system. There were 9 type A fractures (extra-articular), 7 type B fractures (partial articular), and 11 type C fractures (complex articular). 

Six patients had a missing data point at the 6-month mark, and their prior data was carried forward. Seven patients were missing data at the 1-year mark, and their six-month data was carried forward. The carry forward method is a well described method for dealing with missing data points and was determined to be the most appropriate for our data set [5]. One of the patients, who did not return at 1 year, was seen again at 2 years; therefore, there was followup of 1 year or longer on 21 patients (78%). Once all data had been obtained, range of motion, DASH, PRWE scores, and grip strength were analyzed by analysis of variance (ANOVA) and paired Student's *t*-tests.

## 3. Results

**Figure 1:**
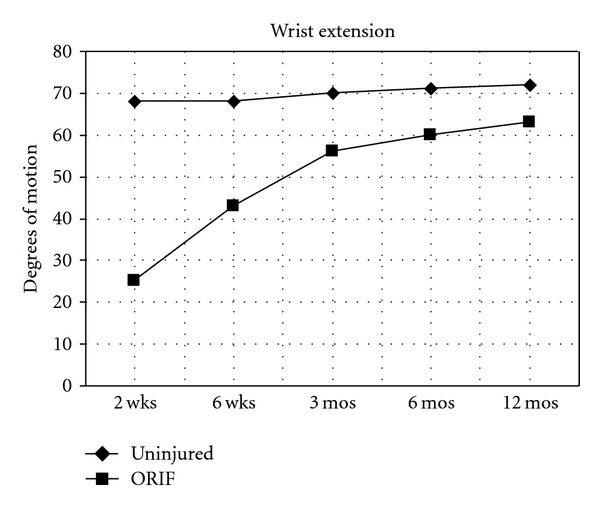
Mean wrist extension measured in degrees at follow-up clinical visits for noninjured wrist and for wrist with an unstable fracture of the distal radius treated with open reduction and internal fixation with a volar fixed-angled plate.

**Figure 2:**
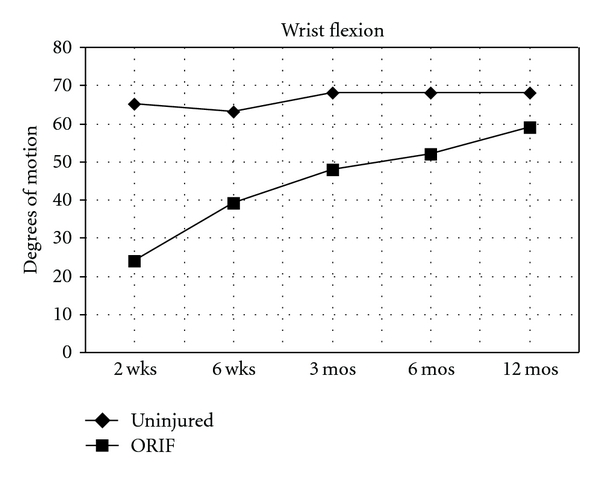
Mean wrist flexion measured in degrees at follow-up clinical visits for noninjured wrist and for wrist with an unstable fracture of the distal radius treated with open reduction and internal fixation with a volar fixed-angled plate.

Ranges of motion measures for the injured and non-injured wrists are shown for each follow-up visit (Table 1). Average measures in degrees are presented with standard deviations. All range of motion parameters showed a trend toward continued improvement out to 12 months. For example, extension in the injured arm was only 25 degrees two weeks after surgery, but rose to 64 degrees by 1 year. Extension, flexion, and supination all improved significantly (*P* < .05) between 2 and 6 weeks, and from 6 weeks until 3 months. Flexion continued to increase, a difference that was statistically significant, also between 3 months and 6 months. Improvement in pronation was statistically significant (*P* < .05) only between 2 weeks and 6 weeks. For all variables we calculated the percent range of motion by using the uninjured wrist as a control. All measures improved between visits as a percentage of the range of motion for the contralateral wrist up to 12 months. At that time, extension in the injured wrist had moved from 37% of the control wrist to 90% (Figure 1). Flexion for the fractured wrist had improved from 37% to 87% of the control wrist (Figure 2). Pronation had improved from 75% to 94% and supination had improved from an initial 52% of the control wrist to 99% by 12 months. 

Our mean DASH scores were 19.1, 17.6, and 14.4 at 3, 6, and 12 months, respectively. This improvement was not statistically significant between any two time points. The PRWE scores were 20.3, 11.4, and 15.6 at 3, 6, and 12 months, respectively. The improvement between 3 months and 6 months was statistically significant (*P* = 0.02) but not between 6 months and 12 months. Grip strength was reported as a percent of the injured side compared to the noninjured side. When the injury was on the dominant side, grip strength was 77% at 12 weeks, 97% at 6 months, and 98% at 1 year. When grip of the injured nondominant side was divided by the dominant side, grip strength was 61% at 12 weeks, 65% at 6 months, and 84% at 1 year.

## 4. Discussion

Over the past decade, there has been a major shift toward operative fixation of displaced distal radius fractures via a volar approach. Only a few prospective studies have documented the rate of return of motion following surgery. Rozental et al. compared ORIF with a volar plate to closed reduction and pin fixation. The main focus was outcome measures but they also reported on wrist range of motion [6]. They found that range of motion and DASH scores improved more quickly in the early post-operative period for the volar plate group; however, by 1 year the scores for the groups were the same. They showed flexion and extension continued to improve out to 1 year, and pronation and supination both returned quickly. They recommended operative fixation for those patients wanting to return quickly to more functional levels of activity. Rozental et al. reported similar results comparing external fixation, radial column plating, and volar plating [6]. Use of a volar plate correlated with improved DASH scores at 3 months, but by 6 months all groups were doing equally well. In their volar plate group, flexion improved out to 6 months and extension continued to improve to 1 year. They also found that pronation returned prior to supination. The main difference we noted was that, despite continued improvement out to 1 year, range of motion never returned to the same level as the uninjured side, except for supination. 

In our study, we focused specifically on rate of recovery noted by return of motion, function, and strength in patients treated with the same volar plate. Extension improved significantly between 2 and 6 weeks and 6 weeks and 3 months (*P* < .05). Flexion showed significant improvement between visits until 6 months. There was some concern that a statistical improvement for later intervals may have been washed out by our carry forward method for missed follow-up appointments. However, a second analysis was performed evaluating only patients with complete data sets and the findings were the same. Pronation improved more rapidly than supination and both returned to near normal at the 1 year time point; supination returned to 99% of the uninjured side.

DASH scores decreased between time points but the mean scores were higher than those reported in other studies [2, 6–9]. The PRWE score, which is more specific for the wrist, improved from the 3 to 6 month time period (*P* = 0.02) but showed no statistically significant improvement afterward. 

Our protocol called for a return visit at 24 months. However, only 11 patients returned at that time—too few for statistical analysis to be meaningful. Also, there seemed to be a negative bias in this data, for patients who were not doing well were more likely to return at 2 years. 

The primary limitation of the study was patient noncompliance with follow-up visits greater than 3 months. This was an unfunded study; therefore, patients were not compensated for returning to clinic. The majority of patients lived a significant distance from our center and were doing well, making it difficult in some cases to convince them to return to our clinic. It is possible that patients returning after 3 months may have created a negative bias; patients whose wrists were not performing to expectation were more likely to return. Having different hand therapists taking the measurements could be considered a limitation. Other limitations include small subject numbers and lack of a comparison group. 

These results should provide answers as surgeons respond to their patients' questions about recovery time following volar plate ORIF for treatment of an unstable distal radius fracture. Greatest gains in motion occur during the first 3 months after surgery; however, all measures continue to show improvement until 1 year. Patients' grip strength, a good indirect measure of function, showed steady improvement. Recovery was more rapid when the dominant side was injured, with grip strength 97% of the contralateral side at 6 months. Grip strength of the injured, nondominant side only reached 84% of the uninjured side at 1 year. Despite the relatively quick return of motion, patients may expect some differences in motion of the injured wrist compared to the contralateral wrist to persist at 12 months.

## Figures and Tables

**Table 1:** Wrist range of motion (degrees ± standard deviations).

		2 Weeks	6 Weeks	3 Months	6 Months	12 Months
Extension	Injured	25 ± 16	43 ± 16*	56 ± 12*	60 ± 12	64 ± 13
	Uninjured	68 ± 9	68 ± 10	70 ± 9	71 ± 10	71 ± 10
	Injured/uninjured	37%	63%	80%	85%	90%

Flexion	Injured	24 ± 13	39 ± 16*	48 ±15*	52 ± 17*	59 ± 15
	Uninjured	66 ± 12	63 ± 14	68 ± 11	68 ± 13	68 ± 11
	Injured/uninjured	37%	61%	71%	76%	87%

Pronation	Injured	63 ± 16	74 ± l2*	78 ± 9	79 ± 10	81 ± 8
	Uninjured	84 ± 5	85 ± 4	84 ± 7	86 ± 5	86 ± 4
	Injured/uninjured	75%	87%	92%	92%	94%

Supination	Injured	41 ± 25	64 ± 20*	74 ± 13*	78 ± 12	80 ± 10
	Uninjured	79 ± 13	80 ± 13	81 ± 14	79 ± 14	80 ± 14
	Injured/uninjured	52%	80%	92%	98%	99%

*P < 0.05, significant improvement compared to prior clinical visit.
